# Prevalence, Clinical Severity, and Serotype Distribution of Pneumococcal Pneumonia Among Adults Hospitalized With Community-Acquired Pneumonia in Tennessee and Georgia, 2018–2022

**DOI:** 10.1093/cid/ciae316

**Published:** 2024-07-17

**Authors:** Wesley H Self, Kelly D Johnson, J Jackson Resser, Cynthia G Whitney, Adrienne Baughman, Mai Kio, Carlos G Grijalva, Jessica Traenkner, Jakea Johnson, Karen F Miller, Christina A Rostad, Inci Yildirim, Luis Salazar, Ralph Tanios, Sydney A Swan, Yuwei Zhu, Jin H Han, Thomas Weiss, Craig Roberts, Nadine Rouphael, For the PNEUMO Study Investigators, Wesley H Self, J Jackson Resser, Adrienne Baughman, Carlos G Grijalva, Jakea Johnson, Karen F Miller, Sydney A Swan, Yuwei Zhu, Jin H Han, Sabrina Shipman, Nadine Rouphael, Cynthia Whitney, Mai Kio, Jessica Traenkner, Christina Rostad, Inci Yildirim, Laurel Bristow, Luis Salazar, Zayna Al-Husein, Evan Anderson, Ifeyinwa K Benyeogor, Andrew Cheng, Jong-Ha Choi, Khalel De Castro, Ana Drobeniuc, Kieffer Hellmeister, Ariel Kay, Matthew Lee, Vikash Patel, Olivia D Reese, Veronica Smith, Ralph Tanios, Elizabeth Grace Taylor, Megan Taylor, Wesley Washington, Cecilia Zhang, Kelly D Johnson, Thomas Weiss, Craig Roberts, Madelyn Ruggieri

**Affiliations:** Vanderbilt Institute for Clinical and Translational Research, Vanderbilt University Medical Center, Nashville, Tennessee, USA; Department of Emergency Medicine, Vanderbilt University Medical Center, Nashville, Tennessee, USA; Value & Implementation, Outcomes Research, Merck Sharp & Dohme, Rahway, New Jersey, USA; Department of Biostatistics, Vanderbilt University Medical Center, Nashville, Tennessee, USA; Global Health Institute and Rollins School of Public Health, Emory University, Atlanta, Georgia, USA; Department of Emergency Medicine, Vanderbilt University Medical Center, Nashville, Tennessee, USA; Department of Medicine, Emory University, Atlanta, Georgia, USA; Department of Health Policy, Vanderbilt University Medical Center, Nashville, Tennessee, USA; Department of Medicine, Emory University, Atlanta, Georgia, USA; Department of Emergency Medicine, Vanderbilt University Medical Center, Nashville, Tennessee, USA; Department of Emergency Medicine, Vanderbilt University Medical Center, Nashville, Tennessee, USA; Department of Pediatrics, Emory University, Atlanta, Georgia, USA; Department of Pediatrics, Yale University School of Medicine, New Haven, Connecticut, USA; Department of Epidemiology of Microbial Diseases, Yale School of Public Health, New Haven, Connecticut, USA; Department of Pediatrics, Emory University, Atlanta, Georgia, USA; Department of Medicine, Emory University, Atlanta, Georgia, USA; Department of Biostatistics, Vanderbilt University Medical Center, Nashville, Tennessee, USA; Department of Biostatistics, Vanderbilt University Medical Center, Nashville, Tennessee, USA; Department of Emergency Medicine, Vanderbilt University Medical Center, Nashville, Tennessee, USA; Geriatric Research, Education, and Clinical Center, Tennessee Valley Healthcare Center, Nashville, Tennessee, USA; Value & Implementation, Outcomes Research, Merck Sharp & Dohme, Rahway, New Jersey, USA; Value & Implementation, Outcomes Research, Merck Sharp & Dohme, Rahway, New Jersey, USA; Department of Medicine, Emory University, Atlanta, Georgia, USA

**Keywords:** *Streptococcus pneumoniae*, pneumococcus, pneumonia, vaccine, serotype

## Abstract

**Introduction:**

Understanding the pneumococcal serotypes causing community-acquired pneumonia (CAP) is essential for evaluating the impact of pneumococcal vaccines.

**Methods:**

We conducted a prospective surveillance study of adults aged ≥18 years hospitalized with CAP at 3 hospitals in Tennessee and Georgia between 1 September 2018 and 31 October 2022. We assessed for pneumococcal etiology with cultures, the BinaxNOW urinary antigen detection test, and serotype-specific urinary antigen detection assays that detect 30 pneumococcal serotypes contained in the investigational pneumococcal conjugate vaccine V116, as well as licensed vaccines PCV15 and PCV20 (except serotype 15B). The distribution of pneumococcal serotypes was calculated based on serotype-specific urinary antigen detection results.

**Results:**

Among 2917 hospitalized adults enrolled with CAP, 352 (12.1%) patients had *Streptococcus pneumoniae* detected, including 51 (1.7%) patients with invasive pneumococcal pneumonia. The 8 most commonly detected serotypes were: 3, 22F, 19A, 35B, 9N, 19F, 23A, and 11A. Among 2917 adults with CAP, 272 (9.3%) had a serotype detected that is contained in V116, compared to 196 (6.7%) patients with a serotype contained in PCV20 (*P* < .001), and 168 (5.8%) patients with a serotype contained in PCV15 (*P* < .001). A serotype contained in V116 but not PCV15 or PCV20 was detected in 120 (4.1%) patients, representing 38.0% of serotype detections.

**Conclusions:**

Approximately 12% of adults hospitalized with CAP had *S. pneumoniae* detected, and approximately one-third of the detected pneumococcal serotypes were not contained in PCV15 or PCV20. Development of new pneumococcal vaccines with expanded serotype coverage has the potential to prevent a substantial burden of disease.

## Teaser

Among 2917 adults hospitalized with community-acquired pneumonia at 3 hospitals in the United States, 352 (12.1%) patients had *Streptococcus pneumoniae* detected and approximately one-third of detected pneumococcal serotypes were not contained in the pneumococcal conjugate vaccines PCV15 or PCV20.

## Graphical Abstract

**Graphical Abstract:**
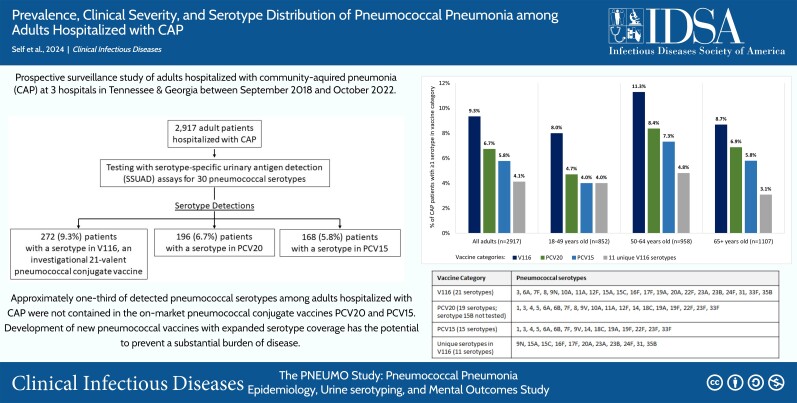
This graphical abstract is also available at Tidbit: https://tidbitapp.io/tidbits/prevalence-clinical-severity-and-serotype-distribution-of-pneumococcal-pneumonia-among-adults-hospitalized-with-community-acquired-pneumonia-in-tennessee-and-georgia-2018-2022

Pneumococcal conjugate vaccines have greatly benefited human health, most notably in the reduction of invasive pneumococcal disease and pneumonia [1–5]. However, despite pneumococcal vaccination programs for both children and adults, *Streptococcus pneumoniae* continues to cause a substantial burden of severe disease in the United States [6–8].

Protection conferred by pneumococcal conjugate vaccines is serotype-specific, meaning a vaccine is designed to prevent disease caused only by the specific pneumococcal serotypes contained within that vaccine [3,9]. Although early investigational work is ongoing for “universal” serotype-independent pneumococcal vaccines, current strategies to improve protection delivered by pneumococcal vaccines focus on expanding the serotypes contained in conjugate vaccines [3,9]. Of the >100 known pneumococcal serotypes [10], vaccine manufacturers have focused on developing conjugate vaccines containing serotypes that cause the greatest burden of human disease [3,9, 11]. A 7-valent (containing 7 serotypes) pneumococcal conjugate vaccine (PCV7) was introduced in the United States in 2000, followed by a 13-valent vaccine (PCV13) in 2010, and a 15-valent vaccine (PCV15) and 20-valent vaccine (PCV20) in 2021 [11]. Current recommendations for PCV use in US adults from the Advisory Committee on Immunization Practices include administration of PCV15 or PCV20 [8].

V116 (Capvaxive; Merck, Sharp & Dohme LLC, a subsidiary of Merck & Co, Inc., Rahway, NJ, USA [MSD]) is a 21-valent pneumococcal conjugate vaccine that was recently approved by the US Food and Drug Administration for adults following clinical trials demonstrating its safety, tolerability, and immunogenicity [12–14]. To assess potential disease coverage for V116 among adults with community-acquired pneumonia (CAP), we conducted a prospective surveillance study to evaluate the prevalence of pneumococcal serotypes among adults hospitalized with CAP that are contained in V116 compared to those in PCV15 and PCV20.

## METHODS

### Study Design

The Pneumococcal Pneumonia Epidemiology, Urine serotyping, and Mental Outcomes (PNEUMO) study is a multicenter, prospective, active surveillance, observational study aimed at understanding the epidemiology of CAP, and specifically pneumococcal CAP, among hospitalized adults. The study was funded by Merck Sharp & Dohme, LLC, the developer of Vaxneuvance (PCV15) and Capvaxive (V116) [12, 13]. The study was coordinated by Vanderbilt University Medical Center and included 3 enrolling sites, including Vanderbilt University Medical Center (Nashville, Tennessee), Emory University Hospital (Atlanta, Georgia), and Emory Midtown Hospital (Atlanta, Georgia). The study was approved by institutional review boards at Vanderbilt University Medical Center and Emory University.

This analysis included patients enrolled between 1 September 2018 and 31 October 2022, which included enrollment for 18 months before onset of COVID-19 in the United States and during the COVID-19 pandemic. A temporary enrollment pause occurred at each site due to restrictions imposed during the COVID-19 pandemic. The enrollment pause at Vanderbilt occurred between 1 September 2020 and 31 October 2020, and at the 2 Emory hospitals between 11 March 2020 and 5 January 2021.

### Participants

Adults hospitalized with CAP were prospectively enrolled. Key eligibility criteria included: age ≥18 years; admitted to the hospital; clinical signs and/or symptoms consistent with an acute respiratory infection; radiographic evidence of pneumonia; and ability to provide a urine sample. Patients who developed pneumonia >48 hours after hospital admission were considered to have hospital-acquired pneumonia and were not eligible for enrollment. The full set of eligibility criteria is shown in Supplementary Appendix B (Supplementary Materials).

### Data Collection

Demographic and clinical data were collected via patient interview, proxy interview when the patient was unable to provide information, and medical record review using standardized data collection forms. The burden of chronic medical conditions was summarized using the Charlson Comorbidity Index [15]. Pneumonia severity at the time of hospital admission was summarized with the CURB-65 score [16]. In-hospital clinical outcomes, including organ support therapies, hospital length of stay, and mortality, were captured from the medical record for the patient's index hospitalization for pneumonia. Definitions for each outcome are detailed in Supplementary Appendix B.

### Pneumococcal Testing

Enrolled patients had a urine sample collected within 72 hours of hospital admission. Urine was tested by research personnel at each site using the BinaxNOW *S. pneumoniae* antigen card (Abbott), which detects the *S. pneumoniae* C-polysaccharide cell wall antigen [17, 18]. This antigen is common to all pneumococcal serotypes; thus, the BinaxNOW test identified the presence of *S. pneumoniae* but did not distinguish among serotypes.

Aliquots of urine from enrolled patients were also shipped to Merck & Co., Inc. (West Point, Pennsylvania, USA), where testing was completed for 30 pneumococcal serotypes using serotype-specific urinary antigen detection (SSUAD) assays [19, 20]. These assays can detect the following 30 pneumococcal serotypes: 1, 3, 4, 5, 6A (cross-reactivity with 6C), 6B, 7F, 8, 9N, 9V, 10A, 11A, 12F, 14, 15A, 15C, 16F, 17F, 18C, 19A, 19F, 20A, 22F, 23A, 23B, 23F, 24F, 31, 33F, and 35B (Supplementary Table 1). This includes all serotypes in PCV15, V116, and PCV20, with the exception of serotype 15B, which is in PCV20. Detection of 1 or 2 pneumococcal serotypes in a sample was considered a positive result. In cases with 2 serotypes detected, both serotypes were reported. Similar to prior studies [21–23], detection of ≥3 serotypes by SSUAD in a sample was deemed an indeterminate result and not reported as evidence of pneumococcal infection. The distribution of pneumococcal serotypes reported in this study was based on SSUAD results only.

Results of bacterial cultures collected for clinical purposes within 48 hours of hospital admission were compiled. A culture positive for *S. pneumoniae* from a normally sterile site—including blood, pleural fluid, cerebrospinal fluid, or synovial fluid—was considered evidence of pneumococcal etiology. A culture positive for *S. pneumoniae* from a respiratory site not normally sterile (sputum, endotracheal aspirate) was considered evidence for pneumococcal etiology if the sample had ≥25 white blood cells/lpf and ≤10 epithelial cells/lpf [6]. *S. pneumoniae* cultured from blood underwent whole genome sequencing to identify pneumococcal serotype (Supplementary Appendix B).

### Community-acquired Pneumonia Categories by *S. pneumoniae* Detection

The study population comprised adults hospitalized with all-cause CAP: that is, community-acquired pneumonia regardless of etiology. Patients within the all-cause CAP population with *S. pneumonia*e detected constituted the pneumococcal CAP population. Patients within the all-cause CAP population without *S. pneumoniae* detected were classified as having nonpneumococcal CAP. Within the pneumococcal CAP population, patients with a positive culture for *S. pneumoniae* from a normally sterile site were classified as invasive pneumococcal CAP. Patients with pneumococcal detection without a positive culture from a normally sterile site were classified as noninvasive pneumococcal CAP.

### Pneumococcal Serotype Categories by Vaccine Type

Pneumococcal serotypes were grouped based on the serotypes contained in PCV15, PCV20, and V116 (Supplementary Table 1). The 11 serotypes contained in V116 but not PCV15 or PCV20 included 9N, 15A, 15C, 16F, 17F, 20A, 23A, 23B, 24F, 31, and 35B.

### Statistical Analyses

Enrolled patients with radiographic evidence of pneumonia and SSUAD testing completed were analyzed. The contribution of each diagnostic test for the detection of *S. pneumoniae* was described. The prevalence of pneumococcal CAP among all-cause CAP was calculated as the number of patients with pneumococcal detection divided by the total number of patients hospitalized with CAP. Prevalence of pneumococcal CAP was calculated for the overall population and for subgroups of interest, including by age group, sex, race, ethnicity, chronic medical conditions, time period (before vs during COVID-19 activity), and presence of a high-risk condition for pneumococcal infection [21] (Supplementary Appendix B).

Baseline characteristics and clinical outcomes were compared between patients with pneumococcal CAP and nonpneumococcal CAP using the chi-square test for categorical variables and the Wilcoxon rank-sum test for continuous variables. The distribution of pneumococcal serotypes detected by SSUAD was reported for the overall population, and by age group, time period, and presence of a high-risk condition for pneumococcal infection. Prevalence of a detected serotype contained in V116 versus PCV15 and V116 versus PCV20 was compared using the chi-square test.

Analyses were completed with R Software, version 4.2.2 (R Foundation, Vienna, Austria; www.r-project.org).

## RESULTS

### Participants

**Figure 1.**
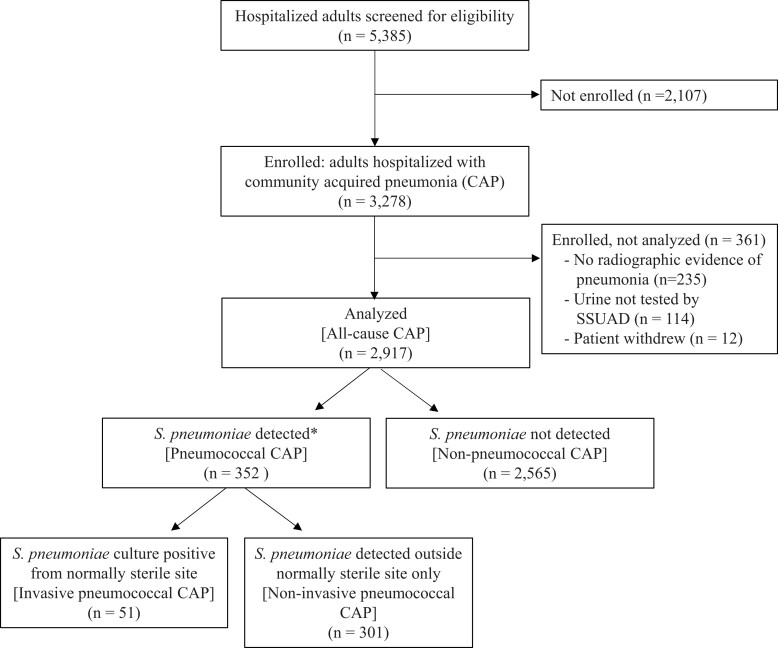
Flow diagram of patient participation. Abbreviations: CAP, community-acquired pneumonia; *S. pneumoniae: Streptococcus pneumoniae;* SSUAD, serotype-specific urinary antigen detection assays. *12 patients who tested positive for ≥3 pneumococcal serotypes by SSUAD and had no other positive tests for *S. pneumoniae* were classified as not having *S. pneumoniae* detected due to risk of false-positive SSUAD results.

Study teams screened 5385 hospitalized adults with a presentation potentially consistent with pneumonia and enrolled 3278 patients. After enrollment, 361 patients were excluded, including 235 patients identified as not having radiographic evidence of pneumonia, 114 patients without SSUAD testing, and 12 patients who withdrew consent, resulting in an analytical population of 2917 patients with all-cause CAP (Figure 1). Among 2917 patients with all-cause CAP, 352 (12.1%) patients had pneumococcal CAP, including 51 patients with invasive pneumococcal CAP (representing 14.5% of pneumococcal CAP) and 301 patients with noninvasive pneumococcal CAP (representing 85.5% of pneumococcal CAP).

Among 2917 patients with all-cause CAP, median age was 60 years, 1970 (67.5%) were White, 847 (29.0%) were Black, 108 (3.7%) were Hispanic, 2702 (92.6%) lived in the community before illness onset, 344 (11.8%) had a high risk CURB-65 score of ≥3, and 1533 (52.6%) were enrolled before onset of COVID-19 activity in the United States (Table 1). Overall, the characteristics of patients with pneumococcal and nonpneumococcal CAP were similar, with notable exceptions including the pneumococcal CAP group having a higher percentage of Black patients (41.2% vs 27.4%; *P* < .001), higher percentage who smoked tobacco regularly (58.7% vs 48.3%; *P* < .001), higher percentage who used alcohol >3 times per week (8.6% vs 5.7%; *P* = .032), higher percentage who interacted with children aged <5 years ≥1 time per week (35.9% vs 27.6%; *P* = .002), higher percentage with chronic obstructive pulmonary disease (28.1% vs 19.3%; *P* < .001), and lower percentage with obesity (26.1% vs 40.7%; *P* < .001).

**Table 1.** Patient Characteristics at Hospital Admission of Adults Hospitalized With Community-acquired Pneumonia, Overall and by Pneumococcal Status

Characteristic	Total [All-Cause CAP] (n = 2917)	*S. Pneumoniae* Detected [Pneumococcal CAP] (n = 352)	*S. Pneumoniae* Not Detected [Nonpneumococcal CAP] (n = 2565)	*P* Value (*S. pneumoniae* Detected vs Not Detected)
Age in years, median (IQR)	60 (47, 70)	60 (51, 70)	61 (47, 70)	.565
Age category, n (%)				.006
18–49 y	852 (29.2%)	85 (24.1%)	767 (29.9%)	
50–64 y	958 (32.8%)	141 (40.1%)	817 (31.9%)	
≥65 y	1107 (37.9%)	126 (35.8%)	981 (38.2%)	
Female sex assigned at birth, n (%)	1314 (45.0%)	167 (47.4%)	1147 (44.7%)	.335
Race, n (%)^a^				<.001
White	1970 (67.5%)	198 (56.2%)	1772 (69.1%)	
Black	847 (29.0%)	145 (41.2%)	702 (27.4%)	
Asian	39 (1.3%)	1 (0.3%)	38 (1.5%)	
American Indian/Native Alaskan	16 (0.5%)	2 (0.6%)	14 (0.5%)	
Native Hawaiian/Pacific Islander	8 (0.3%)	2 (0.6%)	6 (0.2%)	
Other	60 (2.1%)	6 (1.7%)	54 (2.1%)	
Ethnicity, n (%)				.947
Not Hispanic	2722 (93.3%)	328 (93.2%)	2394 (93.3%)	
Hispanic	108 (3.7%)	14 (4.0%)	94 (3.7%)	
Unknown	87 (3.0%)	10 (2.8%)	77 (3.0%)	
Type of home before illness, n (%)				.266
Community dwelling	2702 (92.6%)	320 (90.9%)	2382 (92.9%)	
Nursing home	49 (1.7%)	6 (1.7%)	43 (1.7%)	
Assisted living	39 (1.3%)	6 (1.7%)	33 (1.3%)	
Rehabilitation hospital	14 (0.5%)	1 (0.3%)	13 (0.5%)	
School housing	0 (0.0%)	0 (0.0%)	0 (0.0%)	
Homeless/shelter	52 (1.8%)	12 (3.4%)	40 (1.6%)	
Other	36 (1.2%)	3 (0.9%)	33 (1.3%)	
Unknown	25 (0.9%)	4 (1.1%)	21 (0.8%)	
Ever regularly smoked tobacco, n (%)	1437/2902 (49.5%)	205/349 (58.7%)	1232/2553 (48.3%)	<.001
Alcohol use >3 d/wk, n (%)	175/2890 (6.1%)	30/348 (8.6%)	145/2542 (5.7%)	.032
Use of opioids at least weekly, n (%)	506/2801 (18.1%)	68/339 (20.1%)	438/2462 (17.8%)	.309
Interacts with child aged <5 y at least once per week, n (%)	803/2807 (28.6%)	122/340 (35.9%)	681/2467 (27.6%)	.002
Lives with children, n (%)	600/2880 (20.8%)	83/346 (24.0%)	517/2534 (20.4%)	.123
Received antibiotics for current illness before hospitalization, n (%)	684/2625 (26.1%)	71/328 (21.6%)	613/2297 (26.7%)	.052
Duration of acute illness before hospital admission [d], median (IQR)	2.7 (1.2, 5.7) [32 missing]	2.7 (1.4, 5.1) [5 missing]	2.7 (1.1, 5.7) [27 missing]	.927
Charlson Comorbidity Index, median (IQR) ^b^	4 (2, 6)	4 (2, 6)	4 (2, 6)	.279
Chronic medical conditions, n (%)				
Dementia	78 (2.7%)	12 (3.4%)	66 (2.6%)	.362
COPD	594 (20.4%)	99 (28.1%)	495 (19.3%)	<.001
Asthma	550 (18.9%)	72 (20.5%)	478 (18.6%)	.413
Heart failure	518 (17.8%)	64 (18.2%)	454 (17.7%)	.824
Prior myocardial infarction	268 (9.2%)	37 (10.5%)	231 (9.0%)	.359
Prior stroke	295 (10.1%)	38 (10.8%)	257 (10.0%)	.651
End-stage kidney disease with chronic kidney replacement therapy	141 (4.8%)	14 (4.0%)	127 (5.0%)	.424
Diabetes mellitus	770 (26.4%)	82 (23.3%)	688 (26.8%)	.159
Chronic liver disease	206 (7.1%)	30 (8.5%)	176 (6.9%)	.254
Immunosuppression	612 (21.0%)	74 (21.0%)	538 (21.0%)	.983
Solid organ cancer	681 (23.3%)	82 (23.3%)	599 (23.4%)	.981
Hematologic cancer	228 (7.8%)	30 (8.5%)	198 (7.7%)	.598
Solid organ transplant	231 (7.9%)	25 (7.1%)	206 (8.0%)	.545
Pregnant	22 (0.8%)	0 (0.0%)	22 (0.9%)	.081
Cerebrospinal fluid leak	17/2886 (0.6%)	1/349 (0.3%)	16/2537 (0.6%)	.431
Cochlear implant	29/2901 (1.0%)	3/350 (0.9%)	26/2551 (1.0%)	.775
Obesity with body mass index >30 kg/m^2^	1099/2827 (38.9%)	90/345 (26.1%)	1009/2482 (40.7%)	<.001
CURB-65 score at hospital admission, n (%)^c^				.171
0	979 (33.6%)	109 (31.0%)	870 (33.9%)	
1	971 (33.3%)	126 (35.8%)	845 (32.9%)	
2	623 (21.4%)	65 (18.5%)	558 (21.8%)	
3	280 (9.6%)	40 (11.4%)	240 (9.4%)	
4	61 (2.1%)	11 (3.1%)	50 (1.9%)	
5	3 (0.1%)	1 (0.3%)	2 (0.1%)	
Timing of enrollment				<.001
Before COVID-19 in United States (September 2018–February 2020)	1533 (52.6%)	238 (67.6%)	1295 (50.5%)	
During COVID-19 in United States (March 2020–October 2022)	1384 (47.4%)	114 (32.4%)	1270 (49.5%)	
Location of enrollment				<.001
Tennessee	2164 (74.2%)	217 (61.6%)	1947 (75.9%)	
Georgia	753 (25.8%)	135 (38.4%)	618 (24.1%)	

Abbreviations: CAP, community-acquired pneumonia; COPD, chronic obstructive pulmonary disease; CURB, confusion, uremia, respiratory rate, blood pressure; IQR, interquartile range; *S. pneumoniae, Streptococcus pneumoniae*.

^a^Patients could report multiple races. When patients reported multiple races, they were included in all race categories in this table that they reported.

^b^Charlson comorbidity index was calculated based on the principles described by Charlson et al [15].

^c^The CURB-65 pneumonia severity score was calculated based on the principles described by Lim et al [16].

### Pneumococcal Detection and Prevalence

**Figure 2.**
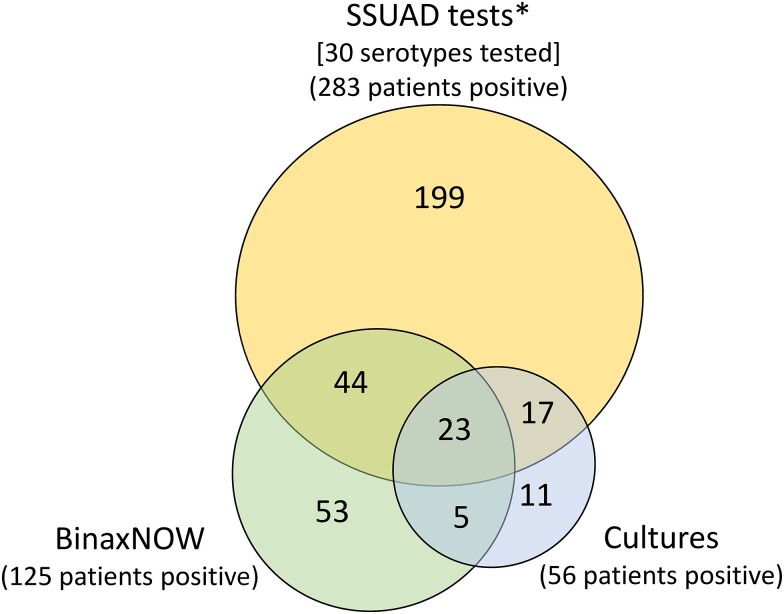
Detection of *S. pneumoniae* by different diagnostic tests among adults hospitalized with pneumococcal community acquired pneumonia. The figure displays counts of patients with different patterns of positive tests for *S. pneumoniae*. These data are shown in tabular form in Supplementary Tables 3 and 4. Abbreviation: SSUAD, serotype-specific urinary antigen detection. *15 patients tested positive for ≥3 pneumococcal serotypes by SSUAD. These patients were classified as negative by SSUAD testing due to risk of false-positive results.

Among 2917 all-cause CAP patients, all underwent SSUAD testing, 2899 (99.4%) had a BinaxNOW *S. pneumoniae* urinary antigen test, and 2224 (76.2%) had ≥1 blood culture completed (Supplementary Table 2). There were 466 positive *S. pneumoniae* tests from 352 unique patients (Figure 2; Supplementary Table 3-4). Among the 466 positive tests, the most common positive test was an SSUAD assay (n = 283; 60.7% of positive tests), followed by BinaxNow antigen test (n = 125; 26.8% of positive tests) and blood culture (n = 49; 10.5% of positive tests). Among the 352 patients with *S. pneumoniae* detected, SSUAD was the only positive test in 199 (56.5%) patients.

Overall, pneumococcal CAP prevalence among all-cause CAP was 12.1%, with higher prevalence before onset of COVID-19 activity than afterwards (15.5% vs 8.2%, *P* < .001) and among patients with ≥1 high-risk condition than without any high-risk conditions (12.7% vs 9.7%, *P* = .040) (Supplementary Table 5).

### Clinical Outcomes

Among 352 patients with pneumococcal CAP, 93 (26.4%) were treated in an intensive care unit, 20 (5.7%) patients died before hospital discharge, and median hospital length of stay among survivors was 4.2 days. In-hospital outcomes among patients with pneumococcal CAP and nonpneumococcal CAP were similar (Table 2).

**Table 2.** In-hospital Clinical Outcomes Among Adults Hospitalized With Community-acquired Pneumonia, Overall and by Pneumococcal Status

Outcome	Total [All-Cause CAP] (n = 2917)	*S. pneumoniae* Detected [Pneumococcal CAP] (n = 352)	*S. pneumoniae* Not Detected [Non-pneumococcal CAP] (n = 2565)	*P* Value (*S. pneumoniae* Detected vs Not Detected)
ICU admission, n (%)	721 (24.7%)	93 (26.4%)	628 (24.5%)	.430
Receipt of noninvasive ventilation or high-flow nasal oxygen, n (%)	408 (14.0%)	50 (14.2%)	358 (14.0%)	.900
Receipt of invasive mechanical ventilation, n (%)	205 (7.0%)	24 (6.8%)	181 (7.1%)	.870
Receipt of vasopressors, n (%)	157 (5.4%)	28 (8.0%)	129 (5.0%)	.032
Receipt of new renal replacement therapy, n (%)	46 (1.6%)	6 (1.7%)	40 (1.6%)	.838
Pleural drainage procedure, n (%)	65 (2.2%)	9 (2.6%)	56 (2.2%)	.656
Death, n (%)	163 (5.6%)	20 (5.7%)	143 (5.6%)	.935
Number of hospital survivors (discharged from hospital alive), n	2754	332	2422	
Hospital length of stay among survivors [d], median (IQR)	4.2 (2.7, 7.9)	4.2 (2.7, 8.1)	4.2 (2.7, 7.8)	.670
Discharge location among survivors, n (%)				.179
Preadmission home	2448 (88.9%)	288 (86.7%)	2160 (89.2%)	
Different home in community	6 (0.2%)	1 (0.3%)	5 (0.2%)	
New nursing home placement	49 (1.8%)	10 (3.0%)	39 (1.6%)	
New assisted living home placement	10 (0.4%)	2 (0.6%)	8 (0.3%)	
Rehabilitation facility	109 (4.0%)	9 (2.7%)	100 (4.1%)	
Transfer to another acute care hospital	8 (0.3%)	2 (0.6%)	6 (0.2%)	
Unknown	50 (1.8%)	6 (1.8%)	44 (1.8%)	
Other	74 (2.7%)	14 (4.2%)	60 (2.5%)	

Abbreviations: CAP, community-acquired pneumonia; ICU, intensive care unit; IQR, interquartile range; *S. pneumoniae, Streptococcus pneumoniae*.

### Pneumococcal Serotype Detections by SSUAD

Among 2917 adults with all-cause CAP, SSUAD assays resulted in no pneumococcal serotype detections in 2619 (89.8%) patients, 1 serotype detection in 250 (8.6%) patients, 2 serotype detections in 33 (1.1%) patients, and ≥3 serotype detections in 15 (0.5%) patients. SSUAD results from the 15 patients with ≥3 serotypes detected were coded as negative for analysis (Supplementary Table 6).

**Figure 3.**
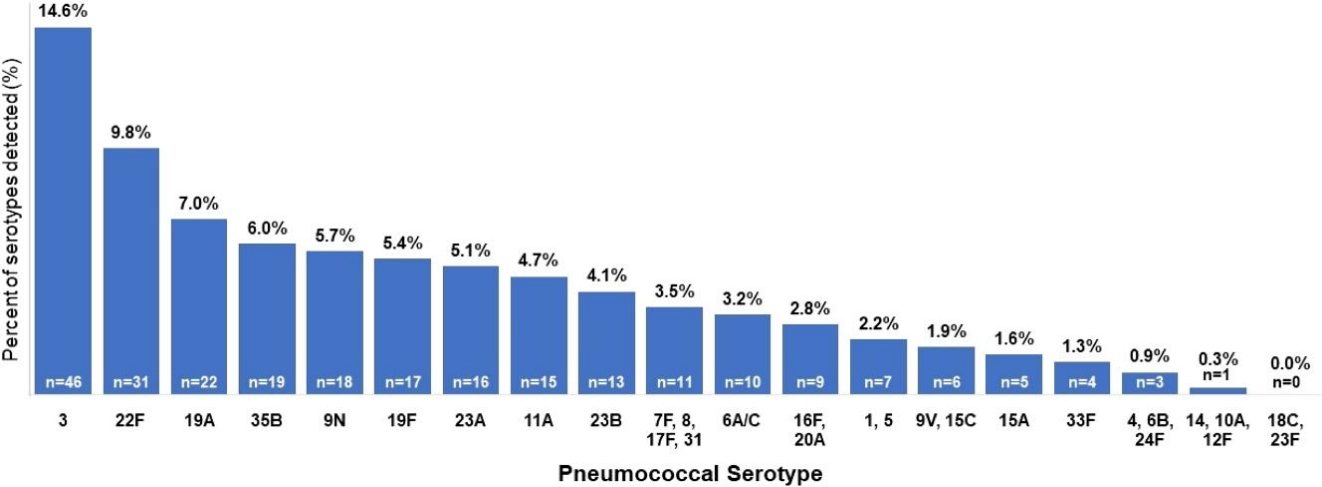
Pneumococcal serotypes detected by SSUAD (30 serotypes evaluated). In total, there were 316 serotype detections. The plot shows the percentage of all serotype detections for each of the 30 serotypes evaluated in this study. The SSUAD assay for serotype 6A has cross-reactivity with serotype 6C.

**Figure 4.**
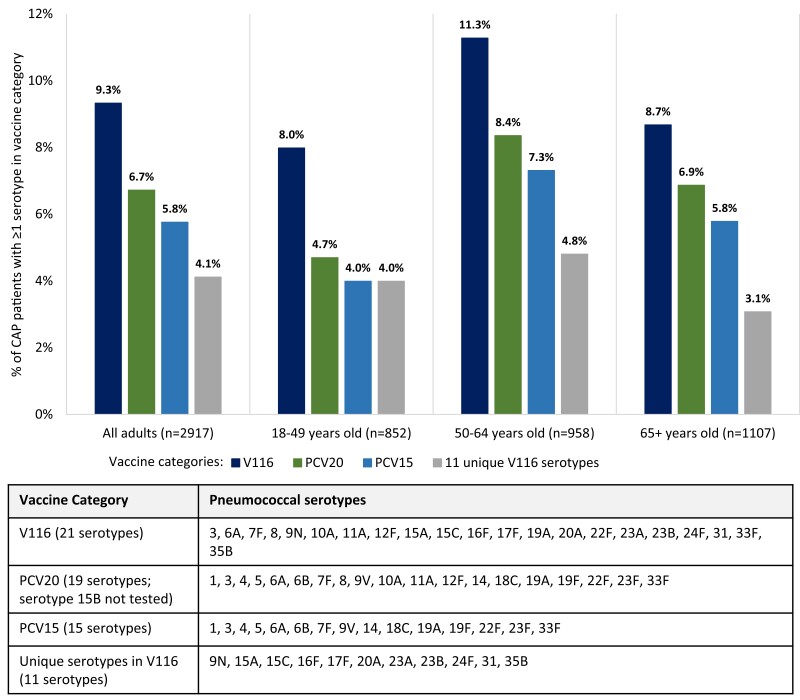
Among patients hospitalized with all-cause community-acquired pneumonia, percentage of patients with pneumococcal serotypes contained within V116, PCV20, and PCV15 overall and by age group. Pneumococcal serotypes detected by SSUAD assays are displayed in these plots. The SSUAD assay for serotype 6A has cross-reactivity with serotype 6C.

Among 2917 adults with all-cause CAP, 283 (9.7%) patients had 1 or 2 pneumococcal serotypes detected by SSUAD assays, with a total of 316 serotype detections among these 283 patients (Figure 3). The 8 most common serotypes were: 3, 22F, 19A, 35B, 9N, 19F, 23A, and 11A (each detected in >0.5% of adults with all-cause CAP) (Supplementary Table 7). Among 2917 adults with all-cause CAP, 272 (9.3%) patients had a serotype detected that is contained in V116 compared to 196 (6.7%) patients with a serotype contained in PCV20 (*P* < .001), and 168 (5.8%) patients with a serotype contained in PCV15 (*P* < .001) (Figure 4). A serotype in V116 but not PCV20 or PCV15 was detected in 120 (4.1%) patients, representing 38.0% of all 316 serotype detections. A serotype not in V116 but in PCV20 and PCV15 was detected in 44 (1.5%) patients, representing 13.9% of all serotype detections.

Pneumococcal serotype distributions stratified by age group, time period, and high-risk conditions are detailed in Supplementary Tables 8–13.

### Comparison of Pneumococcal Serotypes Detected by Blood Culture and SSUAD

Among 2917 all-cause CAP patients, 49 (1.7%) patients had a positive blood culture for *S. pneumoniae.* Among them, 41 patients had a pneumococcal serotype identified from a positive blood culture, and 37 patients had a serotype detected by blood culture that was included in the 30-serotype SSUAD assay set used in this study (Supplementary Table 14). Among these 37 patients, the detected serotype in blood culture was also detected by SSUAD in 31 (83.8%) patients and not detected by SSUAD in 6 (16.2%) patients.

## DISCUSSION

In this study of 2917 adults hospitalized with CAP at 3 US hospitals between 2018 and 2022, approximately 12% of patients were identified as having pneumococcal etiology. Patients with pneumococcal CAP experienced significant morbidity and mortality, with approximately one-quarter treated in an intensive care unit and 6% dying before hospital discharge. These results highlight that *S. pneumoniae* continues to be an important cause of severe pneumonia among adults in the United States more than 20 years after introduction of pneumococcal conjugate vaccines and after COVID-19 altered the epidemiology of respiratory infections [24, 25]. Importantly, approximately one-third of pneumococcal serotypes identified in this study are not included in the 2 pneumococcal conjugate vaccines currently in use (PCV15 and PCV20), suggesting that expansion of serotypes covered by future pneumococcal vaccines could potentially prevent a substantial burden of severe pneumococcal disease.

Development and recent FDA approval of the 21-valent pneumococcal congujate vaccine V116 (Capvaxive) is one effort to expand vaccine coverage to more serotypes [12, 13]. The SSUAD assays used in this study were designed to detect infections with pneumococcal serotypes contained in V116 as well as those in PCV15 and PCV20. In this study, significantly more patients had a pneumococcal serotype that is contained in V116 (9.3%) than in PCV15 (5.8%) or PCV20 (6.7%). These results suggest that V116 may offer a potential advance in covering pneumococcal serotypes causing CAP among US adults. However, none of these vaccines covers all pneumococcal serotypes that cause pneumonia.

The prevalence of pneumococcal etiology among hospitalized US adults with CAP between 2018 and 2022 in this study (12.1%) was similar to the prevalence identified by Isturiz et al [23] (12.3%) among patients hospitalized between 2013 and 2016 using a set of serotype-specific urinary antigen tests for 24 pneumococcal serotypes (Supplementary Table 1). These results are somewhat higher than the prevalence identified in the Etiology of Pneumonia in the Community study [6] (9.7%) that enrolled patients between 2010 and 2012 and used a set of serotype-specific antigen assays for the 13 serotypes in PCV13, reinforcing the concept that expanding the serotypes evaluated by SSUAD incrementally increases the number of hospitalized adults with CAP identified as having pneumococcal disease.

Consistent with prior studies [21, 22, 26], SSUAD assays independently identified a large proportion of pneumococcal cases observed in this study. SSUAD was the only positive pneumococcal test in 56% of pneumococcal cases despite systematic testing with BinaxNOW *S. pneumoniae* urinary antigen tests. Use of SSUAD assays to evaluate pneumococcal etiology has 2 major advantages. First, these assays appear to have increased sensitivity for pneumococcal detection compared to traditional tests, including blood cultures and BinaxNOW urinary antigen tests. Despite only testing for 30 pneumococcal serotypes, the SSUAD assays used in this study identified *S. pneumoniae* in more than twice as many patients as BinaxNOW urinary antigen tests. Second, SSUAD assays provide information on serotype identification for noninvasive pneumococcal pneumonia cases, which is crucial for studying the effectiveness of pneumococcal vaccines and estimating their cost effectiveness.

A potential drawback of using SSUAD assays is the possibility of false-positive results. With any test that increases diagnostic sensitivity compared to historical criterion standards, whether increased positive tests represent true positives or false positives must be carefully considered. The SSUAD assays used in this study were developed by Merck Research Laboratories independently of similar serotype-specific urinary antigen detection assays developed by Pfizer's Vaccine Research and Development Laboratory and used in prior studies [21, 22, 26, 27]. Both sets of assays were calibrated to minimize false-positive results [19, 20, 27], and when applied to clinical populations of adults hospitalized with pneumonia, found similar prevalence of serotypes common to both sets of assays, increasing confidence that positive tests represent true positives [21, 22].

Use of SSUAD assays highlights the concept of simultaneous detection of 2 pneumococcal serotypes in the same patient, which occurred in approximately 1% of patients hospitalized with CAP in this study. The potential for multiple serotypes of *S. pneumoniae* to be identified in a patient's blood has been recognized for decades [28, 29]. Therefore, consistent with prior studies using SSUAD assays [21–23], patients in this study who had 2 serotypes detected by SSUAD were considered positive for both serotypes. The frequency and clinical implications of infection with multiple pneumococcal serotypes is not well understood and the availability of SSUAD assays may now facilitate greater understanding of this phenomenon.

Strengths of this study included use of SSUAD assays to test for 30 pneumococcal serotypes, which is more than prior studies, and consistent methods to enroll patients at multiple sites over a 4-year period, which included time both before and after onset of the COVID-19 pandemic.

This study also had limitations. First, blood and respiratory cultures were not collected from all patients. Results of cultures obtained clinically were reported, but efforts were not made to obtain cultures from all enrolled patients. Prior studies have demonstrated that cultures, even when consistently obtained, yield a small number of pneumococcal cases compared to urinary antigen tests [6, 21]. Second, the SSUAD assay set used in this study did not test for serotype 15B, which is contained in PCV20. Therefore, results of this study may underreport prevalence of PCV20 serotypes. However, prior work demonstrated very low prevalence of serotype 15B [23]. Third, data on prior pneumococcal vaccination for enrolled patients were not captured. Fourth, all study sites were in the southeastern region of the United States and findings may not be fully generalizable to other regions. Fifth, radiographic evidence of pneumonia for study eligibility was based on clinical radiologist interpretation without standardization for study purposes. Sixth, this study was conducted during a period with major shifts in respiratory pathogen epidemiology due to the COVID-19 pandemic [25]. Continued surveillance is needed to understand pneumococcal epidemiology beyond the first 3 years of COVID-19 activity.

## CONCLUSIONS

In this prospective surveillance study conducted in Tennessee and Georgia between 2018 and 2022, approximately 12% of adults hospitalized with CAP had *S. pneumoniae* detected and more than one-third of the detected pneumococcal serotypes were not contained in currently licensed pneumococcal conjugate vaccines (PCV15 and PCV20). Development of new pneumococcal vaccines with expanded serotype coverage has the potential to prevent a substantial burden of disease in adults.

## Supplementary Data

Supplementary materials are available at *Clinical Infectious Diseases* online. Consisting of data provided by the authors to benefit the reader, the posted materials are not copyedited and are the sole responsibility of the authors, so questions or comments should be addressed to the corresponding author.

## Supplementary Material

ciae316_Supplementary_Data

## Notes

***Financial support***. This work was funded by Merck Sharp & Dohme LLC, a subsidiary of Merck & Co., Inc., Rahway, New Jersey, USA. K. D. J., T. W., and C. R. are employees of Merck Sharp & Dohme, LLC. Vanderbilt University Medical Center and Emory University received research funding from Merck Sharp & Dohme LLC to conduct this study.
