# Supplementary material for: Prevalence, Clinical Severity, and Serotype Distribution of Pneumococcal Pneumonia Among Adults Hospitalized With Community-Acquired Pneumonia in Tennessee and Georgia, 2018–2022
Source: Clin Infect Dis. 2024 Jul 17;79(4):838–47. doi: 10.1093/cid/ciae316 (PMC11478805; doi:10.1093/cid/ciae316)
Supplement: ciae316_Supplementary_Data [file ciae316_supplementary_data.docx]

**-- Supplemental Materials --**

**Prevalence, Clinical Severity, and Serotype Distribution of Pneumococcal Pneumonia among Adults Hospitalized with Community Acquired Pneumonia in Tennessee and Georgia, 2018-2022**

Authors: Self WH, Johnson KD, Resser JJ, et al.

Corresponding Author: Wesley H. Self, MD, MPH; email: [wesley.self@vumc.org](mailto:wesley.self@vumc.org)

**Contents of Supplemental Materials:**

[Appendix A. PNEUMO Investigators List 2](#_Toc166939633)

[Appendix B. Supplemental Methods 3](#_Toc166939634)

[1. Eligibility Criteria 3](#_Toc166939635)

[2. Clinical Outcomes 4](#_Toc166939636)

[3. Pneumococcal Whole Genome Sequencing 5](#_Toc166939637)

[4. Subgroups of Interest 6](#_Toc166939638)

[Appendix C. Supplemental Tables 7](#_Toc166939639)

[Table S1. Pneumococcal serotypes in conjugate vaccines and SSUAD assays sets 7](#_Toc166939640)

[Table S2. *S. pneumoniae* testing completed among analyzed patients 9](#_Toc166939641)

[Table S3. Positive *S. pneumoniae* tests group by unique patient 10](#_Toc166939642)

[Table S4. Positive *S. pneumoniae* tests without grouping by unique patient 11](#_Toc166939643)

[Table S5. Prevalence of pneumococcal CAP by subgroup 12](#_Toc166939644)

[Table S6: Description of patients with ≥3 positive SSUAD assays 13](#_Toc166939645)

[Table S7: Pneumococcal serotype distribution among all analyzed patients 14](#_Toc166939646)

[Table S8: Pneumococcal serotype distribution in age group 18-49 years old 15](#_Toc166939647)

[Table S9: Pneumococcal serotype distribution in age group 50-64 years old 16](#_Toc166939648)

[Table S10: Pneumococcal serotype distribution in age group ≥65 years old 17](#_Toc166939649)

[Table S11: Pneumococcal serotype distribution before COVID-19 onset 18](#_Toc166939650)

[Table S12: Pneumococcal serotype distribution during COVID-19 pandemic 19](#_Toc166939651)

[Table S13: Pneumococcal serotype distribution among patients with high-risk conditions 20](#_Toc166939652)

[Table S14: Pneumococcal serotype detections by blood culture and SSUAD 21](#_Toc166939653)

# Appendix A. PNEUMO Investigators List

The investigators and collaborators for the Pneumococcal Pneumonia Epidemiology, Urine Serotyping, and Mental Outcomes (PNEUMO) Study are listed below.

**Vanderbilt University Medical Center**

Wesley H. Self; J. Jackson Resser; Adrienne Baughman; Carlos G. Grijalva; Jakea Johnson; Karen F. Miller; Sydney A. Swan; Yuwei Zhu; Jin H. Han; Sabrina Shipman

**Emory University**

Nadine Rouphael; Cynthia Whitney; Mai Kio; Jessica Traenkner; Christina Rostad; Inci Yildirim; Laurel Bristow; Luis Salazar; Zayna Al-Husein; Evan Anderson; Ifeyinwa K Benyeogor; Andrew Cheng; Jong-Ha Choi; Khalel De Castro; Ana Drobeniuc; Kieffer Hellmeister; Ariel Kay; Matthew Lee; Vikash Patel; Olivia D Reese; Veronica Smith; Ralph Tanios; Elizabeth Grace Taylor; Megan Taylor; Wesley Washington; Cecilia Zhang

**Merck Sharp & Dohme, LLC**

Kelly D. Johnson; Thomas Weiss; Craig Roberts; Madelyn Ruggieri

# Appendix B. Supplemental Methods

## Eligibility Criteria

The inclusion and exclusion criteria for the pneumonia cohort of the PNEUMO study are shown below.

Inclusion Criteria

- - - 1. Age ≥ 18 years old.
      2. Hospitalized, either in in-patient or observation status.
      3. Clinical signs and/or symptoms of an acute respiratory illness, as defined by ≥ 1 of the following:
- New or worsening shortness of breath in the past 7 days
- New or worsening cough in the past 7 days
- New or worsening sputum production in the past 7 days
- New or worsening chest pain in the past 7 days
- Tachypnea to respiratory rate ≥ 22 breaths/min not known to be chronic
- Hypoxia to SpO2 ≤ 92% not known to be chronic
- Initiation of invasive or non-invasive mechanical ventilation
  - - 1. Clinical signs and/or symptoms of an acute infection, as defined by ≥ 1 of the following:
- Body temperature ≥ 38º C (100.4º F) or ≤ 35.5º C (95.9º F)
- Reported fever, chills, or feeling feverish at home without explicit documentation of a fever within the past 7 days
- White blood cell count > 10.7 or < 3.9 thousand cells/mcL
- C-reactive protein > 10 mg/L
- Altered mental status
  - - 1. Radiologic evidence of pneumonia interpreted by a radiologist, defined as ≥ 1 of the following findings on chest x-ray (CXR) or computed tomography (CT) completed within 48 hours before or after hospital admission:
- Pulmonary infiltrate not known to be chronic
- Pulmonary opacity not known to be chronic (including ground glass opacities on CT)
- Pulmonary consolidation not known to be chronic

Exclusion Criteria

- - - 1. Enrollment in this study within the past 30 days.
      2. Development of pneumonia (as defined in the inclusion criteria) more than 48 hours after hospital admission (pneumonia developed 48 hours after admission will be considered hospital acquired-pneumonia).
      3. Inability to obtain consent from patient or surrogate for this study within **72** hours of hospital admission (including time at a transferring hospital prior to admission at a study hospital).
      4. Inability or unwillingness of the patient to provide a urine sample within **72** hours of hospital admission (including time at a transferring hospital prior to admission at a study hospital).
      5. Non-pneumonia illness completely explains the patient’s acute symptoms. Alternative non-pneumonia illnesses could include: pulmonary embolism, acute heart failure, lung cancer, and pulmonary hemorrhage.

Eligibility Notes

After emergence of COVID-19, patients who tested positive for SARS-CoV-2 were considered for enrollment at Vanderbilt University Medical Center (November 1, 2020 – October 31, 2022). Patients who tested positive for SARS-CoV-2 were not considered for enrollment at the Emory University Hospitals.

## Clinical Outcomes

Trained study personnel captured information from the medical record for the following clinical outcomes. Outcome ascertainment was limited to the index hospitalization for pneumonia; events that occurred after hospital discharged for the visit in which the patient was enrolled in the study were not considered in outcome ascertainment.

In-hospital outcomes included:

- - - - 1. Intensive care unit (ICU) admission: treatment for any duration of time in a location within one of the enrolling hospitals designated as an ICU based on local practice. ICUs had the capability of routinely caring for patients on invasive mechanical ventilation.
        2. Receipt of high-flow nasal oxygen: application of supplemental oxygen therapy to the nose at a flow rate ≥30 liters/minute.
        3. Receipt of non-invasive ventilation: application of a mask to the nose or nose and mouth that applies positive pressure ventilation. Bilevel positive airway pressure (BiPAP) and continuous positive airway pressures (CPAP) are two common forms of non-invasive ventilation.
        4. Receipt of invasive mechanical ventilation: application of positive pressure ventilation via a mechanical ventilator through an endotracheal tube or tracheostomy tube.
        5. Receipt of vasopressors: continuous infusion of a medication with the goal of increasing blood pressure. The following medications were considered vasopressors: norepinephrine, epinephrine, dopamine, phenylephrine or vasopressin.
        6. Receipt of new renal replacement therapy: initiation of renal replacement therapy in the hospital for a patient who was not on chronic renal replacement therapy prior to hospital admission. Renal replacement therapy could include intermittent hemodialysis or continuous filtration techniques.
        7. Receipt of a pleural drainage procedure: medical procedure conducted in the hospital to drain fluid from the pleural space. A pleural drainage procedure could include a bedside procedure, such as a thoracentesis, or a procedure in an operating room, such as a video-assisted thoracoscopic surgery (VATS).
        8. Death: patient died in the hospital prior to hospital discharge.
        9. ICU length of stay among survivors: among patients who survived until departure from the ICU, the length of time between initial admission to the ICU and departure from the ICU.
        10. Hospital length of stay among survivors: among patients who survived to hospital discharge, the length of time between initial hospital arrival and hospital discharge.
        11. Discharge location among survivors: categorical variable that described the location where the patient moved after discharge from the hospital. Prespecified categories for this variable included the following:

Pre-admission home

Different home in community

New nursing home placement

New assisted living home placement

Rehabilitation facility

Transfer to another acute care hospital

Unknown

Other

## Pneumococcal Whole Genome Sequencing

*S. pneumoniae* cultured from blood underwent whole genome sequencing to identify pneumococcal serotype. Sequencing was performed in two laboratories with extensive experience in the technique. Culture-positive isolates from Vanderbilt were sequenced at JMI Laboratories (<https://www.jmilabs.com/>) and isolates from Emory hospitals were sequenced at the US Centers for Disease Control and Prevention (CDC) in the laboratory where sequencing for the Active Bacterial Core Surveillance (ABCs) is performed ([ABCs Laboratory Characterization | CDC](https://www.cdc.gov/abcs/methodology/lab-characterization.html)).

## Subgroups of Interest

Subgroups of interest in this study included:

| **Category** | **Groups** |
| --- | --- |
| By Age Group | |
|  | 18-49 years old |
|  | 50-64 years old |
|  | ≥65 years old |
| By sex | |
|  | Female |
|  | Male |
| By race | |
|  | Black |
|  | White |
| By ethnicity | |
|  | Hispanic |
|  | Not Hispanic |
| By presence of chronic medical conditions | |
|  | Immunosuppression present vs absent |
|  | COPD or asthma present vs absent |
|  | Diabetes mellitus present vs absent |
| By study year | |
|  | Year 1: September 2018 – August 2019 |
|  | Year 2: September 2019 – August 2020 |
|  | Year 3: November 2020 – October 2021 |
|  | Year 4: November 2021 – October 2022 |
| By timing of COVID-19 onset in the US | |
|  | Before COVID-19 in US (September 2018 – February 2020) |
|  | After COVID-19 in US (March 2020 – October 2022) |
| By presence of ≥1 high risk condition for pneumococcal infection as defined by US Centers for Disease Control and Prevention (CDC)^a^ | |
|  | High risk, defined as presence of ≥1 of the following:   - Alcoholism - Cigarette smoking - Cerebrospinal fluid leak - Chronic heart disease (congestive heart failure and cardiomyopathies) - Chronic liver disease - Chronic lung disease, including chronic obstructive pulmonary disease, emphysema, and asthma - Cochlear implant - Immunosuppression - Diabetes mellitus |
|  | Not high-risk, defined as having none of the conditions listed above. |

High risk conditions for pneumococcal infection identified by CDC; accessed from the following CDC website on February 18, 2024: <https://www.cdc.gov/vaccines/vpd/pneumo/hcp/who-when-to-vaccinate.html>

# Appendix C. Supplemental Tables

## Table S1. Pneumococcal serotypes in conjugate vaccines and SSUAD assays sets

This table shows the pneumococcal serotypes contained in the pneumococcal conjugate vaccines PCV15, PCV20, and V116 and sets of serotype-specific urinary antigen detection (SSUAD) assays used in prior studies and this study.

|  | **Pneumococcal Conjugate Vaccines** | | | **Sets of SSUAD Assays** | | |
| --- | --- | --- | --- | --- | --- | --- |
| **Pneumococcal serotype** | **PCV15**  **(15 valent vaccine)** | **PCV20**  **(20 valent vaccine)** | **V116**  **(21 valent vaccine)** | **13 serotype set ^a^** | **24 serotype set ^b^** | **30 serotype set (used in this study) ^c^** |
| 1 | ✓ | ✓ |  | ✓ | ✓ | ✓ |
| 2 |  |  |  |  | ✓ |  |
| 3 | ✓ | ✓ | ✓ | ✓ | ✓ | ✓ |
| 4 | ✓ | ✓ |  | ✓ | ✓ | ✓ |
| 5 | ✓ | ✓ |  | ✓ | ✓ | ✓ |
| 6A ^d^ | ✓ | ✓ | ✓ | ✓ | ✓ | ✓ |
| 6B | ✓ | ✓ |  | ✓ | ✓ | ✓ |
| 7F | ✓ | ✓ | ✓ | ✓ | ✓ | ✓ |
| 8 |  | ✓ | ✓ |  | ✓ | ✓ |
| 9N |  |  | ✓ |  | ✓ | ✓ |
| 9V | ✓ | ✓ |  | ✓ | ✓ | ✓ |
| 10A |  | ✓ | ✓ |  | ✓ | ✓ |
| 11A |  | ✓ | ✓ |  | ✓ | ✓ |
| 12F |  | ✓ | ✓ |  | ✓ | ✓ |
| 14 | ✓ | ✓ |  | ✓ | ✓ | ✓ |
| 15A |  |  | ✓ |  |  | ✓ |
| 15B ^e^ |  | ✓ |  |  | ✓ |  |
| 15C |  |  | ✓ |  |  | ✓ |
| 16F |  |  | ✓ |  |  | ✓ |
| 17F |  |  | ✓ |  | ✓ | ✓ |
| 18C | ✓ | ✓ |  | ✓ | ✓ | ✓ |
| 19A | ✓ | ✓ | ✓ | ✓ | ✓ | ✓ |
| 19F | ✓ | ✓ |  | ✓ | ✓ | ✓ |
| 20A |  |  | ✓ |  | ✓ | ✓ |
| 22F | ✓ | ✓ | ✓ |  | ✓ | ✓ |
| 23A |  |  | ✓ |  |  | ✓ |
| 23B |  |  | ✓ |  |  | ✓ |
| 23F |  |  |  | ✓ |  | ✓ |
| 24F | ✓ | ✓ | ✓ |  | ✓ | ✓ |
| 31 |  |  | ✓ |  |  | ✓ |
| 33F | ✓ | ✓ | ✓ |  | ✓ | ✓ |
| 35B |  |  | ✓ |  |  | ✓ |

Footnotes for Table S1:

- - - - 1. A 13-serotype set of SSAUD assays was developed by Pfizer’s Vaccine Research and Development Laboratory to evaluate for the serotypes in PCV13. This set of assays was used in the following studies: [1] Wunderink et al. Clin Infect Dis 2018; 66(10):1540-10. [2] Isturiz et al. Vaccine. 2019; 37: 3352-3361.
        2. A 24 -serotype set of SSUAD assays was developed by Pfizer’s Vaccine Research and Development Laboratory to evaluate for the serotypes in PCV20 plus 4 additional serotypes. This set of assays was used in the following study: Isturiz et al. Clin Infect Dis 2021; 73(7):1216-22.
        3. A 30-serotype set of SSUAD assays was developed by Merck Research Laboratories to evaluate for the serotypes in PCV15 and V116. This 30-serotype set of assays was used in this study.
        4. Within the 30-serotype set of SSUAD assays, the assay for serotype 6A has cross-reactivity with serotype 6C.
        5. Pneumococcal serotype 15B is contained in PCV20 but not tested by the 30-serotype set of SSUAD assays used in this study. Serotype 15B is the only serotype in PCV15, PCV20, or V116 not tested by SSUAD in this study. The serotype 15B assay used in the 24 serotype set is reported to have cross-reactivity with serotype 15C [Kalina et al, Clinical Infectious Diseases 2020;71(9):e430–8].

## Table S2. *S. pneumoniae* testing completed among analyzed patients

| ***S. pneumoniae* test** | **Patients with this *S. pneumoniae* test completed, n (% of 2917 total patients)** |
| --- | --- |
| SSUAD (30-serotype set) | 2917 (100.0%) |
| BinaxNOW | 2899 (99.4%) |
| Blood culture | 2224 (76.2%) |
| Endotracheal culture | 189 (6.5%) |
| Sputum culture | 324 (11.1%) |
| Synovial fluid culture | 96 (3.3%) |
| Pleural fluid culture | 25 (0.9%) |
| Bronchoalveolar (BAL) fluid culture | 22 (0.8%) |
| Cerebral spinal fluid (CSF) culture | 9 (0.3%) |

## Table S3. Positive *S. pneumoniae* tests group by unique patient

Tabulation of positive *S. pneumoniae* tests, using unique patients with at least one positive *S. pneumoniae* test as the unit of analysis. Overall, there were 466 positive tests for *S. pneumoniae* among 352 unique patients. Table S3a shows each culture type separately while Table S3b collapses cultures into one category. The information in Table S3b is displayed in Figure 2 in the main text.

Table S3a: Each culture type reported separately

| **Number of positive *S. pneumonia tests* in unique patient** | **Positive *S. pneumoniae* test(s)** | **Patients with this pattern of positive *S. pneumoniae* tests, n (% of 352 patients with at least one positive *S. pneumoniae* test)** |
| --- | --- | --- |
| 1 | SSUAD (30 serotype set) | 199 (56.5%) |
|  | BinaxNOW only | 53 (15.1%) |
|  | Blood culture only | 8 (2.3%) |
|  | Synovial fluid only | 1 (0.3%) |
|  | Pleural fluid only | 1 (0.3%) |
|  | Sputum culture only | 1 (0.3%) |
| 2 | SSUAD + BinaxNOW | 44 (12.5%) |
|  | SSUAD + blood culture | 15 (4.3%) |
|  | BinaxNOW + blood culture | 5 (1.4%) |
|  | SSUAD + endotracheal culture | 1 (0.3%) |
|  | SSUAD + sputum culture | 1 (0.3%) |
| 3 | SSUAD + BinaxNOW + blood culture | 19 (5.4%) |
|  | SSUAD + BinaxNOW + endotracheal | 1 (0.3%) |
|  | BinaxNOW + SSUAD + sputum | 1 (0.3%) |
| 4 | SSUAD + BinaxNOW + blood culture + endotracheal culture | 2 (0.6%) |
|  |  |  |
| **Number of patients with ≥ 1 *S. pneumoniae* test positive** | | **352** |

Table S3b: Cultures collapsed into one category

| **Number of positive *S. pneumonia tests* in unique patient** | **Positive *S. pneumoniae* test(s)** | **Patients with this pattern of positive *S. pneumoniae* tests, n (% of 352 patients with at least one positive *S. pneumoniae* test)** |
| --- | --- | --- |
| 1 | SSUAD (30 serotype set) | 199 (56.5%) |
|  | BinaxNOW only | 53 (15.1%) |
|  | Culture only | 11 (3.1%) |
| 2 | SSUAD + BinaxNOW | 44 (12.5%) |
|  | SSUAD + Culture | 17 (4.8%) |
|  | BinaxNOW + Culture | 5 (1.4%) |
| 3 | SSUAD + BinaxNOW + Culture | 23 (6.8%) |
|  |  |  |
| **Number of patients with ≥ 1 *S. pneumoniae* test positive** | | **352** |

## Table S4. Positive *S. pneumoniae* tests without grouping by unique patient

Tabulation of positive *S. pneumoniae* tests, using each positive test as the unit of analysis. Overall, there were 466 positive tests for *S. pneumoniae* among 352 unique patients. Table S4a shows each culture type separately while Table S4b collapses cultures into one category.

Table S4a: Each culture type reported separately

| **Positive *S. pneumoniae* test** | **Patients with this *S. pneumoniae* tests positive, n (% of 466 total positive *S. pneumoniae* tests)** |
| --- | --- |
| SSUAD (30 serotype set) | 283 (60.7%) |
| BinaxNOW | 125 (26.8%) |
| Blood culture | 49 (10.5%) |
| Endotracheal culture | 4 (0.9%) |
| Sputum culture | 3 (0.6%) |
| Synovial fluid culture | 1 (0.2%) |
| Pleural fluid culture | 1 (0.2%) |
| Bronchoalveolar (BAL) fluid culture | 0 (0.0%) |
| Cerebral spinal fluid (CSF) culture | 0 (0.0%) |
|  |  |
| **Number of positive *S. pneumoniae* tests** | **466** |

Table S4b: Cultures collapsed into one category

| **Positive *S. pneumoniae* test** | **Patients with this *S. pneumoniae* tests positive, n (% of 466 total positive *S. pneumoniae* tests)** |
| --- | --- |
| SSUAD (30 serotype set) | 283 (60.7%) |
| BinaxNOW | 125 (26.8%) |
| Blood culture (any type) | 58 (12.4%) |
|  |  |
| **Number of positive *S. pneumoniae* tests** | **466** |

## Table S5. Prevalence of pneumococcal CAP by subgroup

This table displays the prevalence of pneumococcal community acquired pneumonia (CAP) among adults hospitalized with all-cause CAP, overall and by subgroups.

| **Category / Group** | | **Count of pneumococcal CAP (n)** | **Count of all-cause CAP (n)** | **Prevalence of pneumococcal CAP among all-cause CAP (%)** | **P-value ^a^**  **(difference across groups in category)** |
| --- | --- | --- | --- | --- | --- |
| All enrolled patients | | 352 | 2917 | 12.1% |  |
| By age group | |  |  |  | 0.006 |
|  | 18-49 years | 85 | 852 | 10.0% |  |
|  | 50-64 years | 141 | 958 | 14.7% |  |
|  | ≥65 years | 126 | 1107 | 11.4% |  |
| By sex | |  |  |  | 0.365 |
|  | Female | 167 | 1314 | 12.7% |  |
|  | Male | 185 | 1603 | 11.5% |  |
| By Black and White race ^b^ | |  |  |  | <0.001 |
|  | Black | 145 | 843 | 17.2% |  |
|  | White | 198 | 1966 | 10.1% |  |
| By ethnicity | |  |  |  | 0.893 |
|  | Hispanic | 14 | 108 | 13.0% |  |
|  | Not Hispanic | 328 | 2722 | 12.0% |  |
| By immunosuppression status | |  |  |  | 1.000 |
|  | Immunosuppression present | 74 | 612 | 12.1% |  |
|  | Immunosuppression absent | 278 | 2305 | 12.1% |  |
| By COPD/asthma status | |  |  |  | 0.002 |
|  | COPD or asthma present | 139 | 941 | 14.8% |  |
|  | COPD and asthma absent | 213 | 1976 | 10.8% |  |
| By diabetes mellitus status | |  |  |  | 0.179 |
|  | Diabetes mellitus present | 82 | 770 | 10.6% |  |
|  | Diabetes mellitus absent | 270 | 2147 | 12.6% |  |
| By study year | |  |  |  | <0.001 |
|  | Sept 2018 – Aug 2019 | 149 | 964 | 15.5% |  |
|  | Sept 2019 – Aug 2020 | 111 | 771 | 14.4% |  |
|  | Nov 2020 – Oct 2021 | 49 | 648 | 7.6% |  |
|  | Nov 2021 – Oct 2022 | 43 | 534 | 8.1% |  |
| By timing of COVID-19 onset | |  |  |  | <0.001 |
|  | Before COVID-19 in US (Sept 2018 – Feb 2020) | 238 | 1533 | 15.5% |  |
|  | During COVID-19 in US (March 2020 – Oct 2022) | 114 | 1384 | 8.2% |  |
| By presence of a high-risk condition for pneumococcal infection ^c^ | |  |  |  | 0.040 |
|  | ≥1 high-risk condition | 290 | 2275 | 12.7% |  |
|  | No high-risk conditions | 62 | 642 | 9.7% |  |

Footnotes for Table S5

- 1. P values were calculated via the chi-square test with Yates continuity correction.
  2. Patients who reported both Black and White race were excluded from this analysis comparing patients with Black versus White race.
  3. High-risk conditions for pneumococcal infection: alcoholism; cigarette smoking; cerebrospinal fluid leak; chronic heart disease (congestive heart failure and cardiomyopathies); chronic liver disease; chronic lung disease, including chronic obstructive pulmonary disease, emphysema, and asthma; cochlear implant; immunosuppression; diabetes mellitus.

## Table S6: Description of patients with ≥3 positive SSUAD assays

Of the 15 patients with ≥3 SSUAD assays positive:

- 3 (20%) had a positive BinaxNow pneumococcal urinary antigen test
- 1 (6.7%) had a positive blood culture for *S. pneumoniae*

Test results for each of the 15 patients with ≥3 SSUAD assays positive:

| Patient Number | SSUAD assays result | BinaxNow pneumococcal antigen test result | Blood culture result |
| --- | --- | --- | --- |
| 1 | 3, 10A, 17F | Negative | Negative |
| 2 | 3, 19F, 23F | **Positive** | Negative |
| 3 | 3, 7F, 19F | Negative | Negative |
| 4 | 3, 7F, 9N, 10A, 12F | Negative | Negative |
| 5 | 3, 33F, 14, 19F, 23F | Negative | Negative |
| 6 | 3, 7F, 10A | Negative | Negative |
| 7 | 6A/C, 8, 17F | Negative | Negative |
| 8 | 19A, 33F, 5 | Negative | Negative |
| 9 | 3, 6A/C, 1, 4, 5, 14 | **Positive** | Negative |
| 10 | 6A/C, 5, 9V | Negative | Negative |
| 11 | 6A/C, 5, 9V | Negative | Negative |
| 12 | 6A/C, 7F, 19A, 5 | Negative | Negative |
| 13 | 6A/C, 5, 9V, 14 | Negative | Negative |
| 14 | 3, 14, 18C, 19F | Negative | Negative |
| 15 | 3, 6A/C, 7F, 5, 9V | **Positive** | **Positive**  **(*S. pneumoniae*; serotype not identified)** |

## Table S7: Pneumococcal serotype distribution among all analyzed patients

Detection of pneumococcal serotypes by SSUAD assays among adults hospitalized with community-acquired pneumonia. A total of 316 pneumococcal serotype detections occurred in 283 unique patients. This table displays the distribution of pneumococcal serotype detections, with serotypes grouped according to the serotypes contained within three pneumococcal conjugate vaccines (PCV15, PCV20, and V116).

| **Adults Hospitalized with CAP (all analyzed patients)** | | | | |
| --- | --- | --- | --- | --- |
| Conjugate vaccines that include this serotype | Pneumococcal serotype | Frequency count of serotype detection, n | Percentage of pneumococcal serotype detections (denominator= 316) due to this serotype (%) | Percentage of all-cause CAP patients (denominator= 2,917) with this serotype detected (%) |
| PCV15, PCV20 & V116 | 3 | 46 | 14.6% | 1.6% |
|  | 6A ^a^ | 10 | 3.2% | 0.3% |
|  | 7F | 11 | 3.5% | 0.4% |
|  | 19A | 22 | 7.0% | 0.8% |
|  | 22F | 31 | 9.8% | 1.1% |
|  | 33F | 4 | 1.3% | 0.1% |
| PCV15 & PCV20  (not V116) | 1 | 7 | 2.2% | 0.2% |
|  | 4 | 3 | 0.9% | 0.1% |
|  | 5 | 7 | 2.2% | 0.2% |
|  | 6B | 3 | 0.9% | 0.1% |
|  | 9V | 6 | 1.9% | 0.2% |
|  | 14 | 1 | 0.3% | <0.1% |
|  | 18C | 0 | 0.0% | 0.0% |
|  | 19F | 17 | 5.4% | 0.6% |
|  | 23F | 0 | 0.0% | 0.0% |
| PCV20 & V116  (not PCV15) | 8 | 11 | 3.5% | 0.4% |
|  | 10A | 1 | 0.3% | <0.1% |
|  | 11A | 15 | 4.7% | 0.5% |
|  | 12F | 1 | 0.3% | <0.1% |
| V116 only  (not PCV15, not PCV20) | 9N | 18 | 5.7% | 0.6% |
|  | 15A | 5 | 1.6% | 0.2% |
|  | 15C | 6 | 1.9% | 0.2% |
|  | 16F | 9 | 2.8% | 0.3% |
|  | 17F | 11 | 3.5% | 0.4% |
|  | 20A | 9 | 2.8% | 0.3% |
|  | 23A | 16 | 5.1% | 0.5% |
|  | 23B | 13 | 4.1% | 0.4% |
|  | 24F | 3 | 0.9% | 0.1% |
|  | 31 | 11 | 3.5% | 0.4% |
|  | 35B | 19 | 6.0% | 0.7% |
| **Total number of serotype detections** | | **316** |  |  |

- - - - 1. The SSUAD assay for serotype 6A has cross-reactivity with serotype 6C.

## Table S8: Pneumococcal serotype distribution in age group 18-49 years old

Detection of pneumococcal serotypes by SSUAD assays among adults 18-49 years old hospitalized with community-acquired pneumonia. A total of 74 pneumococcal serotypes were detected in 69 unique patients in this age stratum.

| **Age Group: 18 – 49 years old** | | | | |
| --- | --- | --- | --- | --- |
| Conjugate vaccines that include this serotype | Pneumococcal serotype | Patients with serotype detected, n | Percentage of pneumococcal serotype detections (denominator= 74) due to this serotype (%) | Percentage of all-cause CAP patients (denominator= 852) with this serotype detected (%) |
| PCV15, PCV20 & V116 | 3 | 9 | 12.2% | 1.1% |
|  | 6A ^a^ | 2 | 2.7% | 0.2% |
|  | 7F | 1 | 1.4% | 0.1% |
|  | 19A | 8 | 10.8% | 0.9% |
|  | 22F | 7 | 9.5% | 0.8% |
|  | 33F | 1 | 1.4% | 0.1% |
| PCV15 & PCV20  (not V116) | 1 | 0 | 0.0% | 0.0% |
|  | 4 | 0 | 0.0% | 0.0% |
|  | 5 | 2 | 2.7% | 0.2% |
|  | 6B | 0 | 0.0% | 0.0% |
|  | 9V | 3 | 4.1% | 0.4% |
|  | 14 | 0 | 0.0% | 0.0% |
|  | 18C | 0 | 0.0% | 0.0% |
|  | 19F | 1 | 1.4% | 0.1% |
|  | 23F | 0 | 0.0% | 0.0% |
| PCV20 & V116  (not PCV15) | 8 | 3 | 4.1% | 0.4% |
|  | 10A | 0 | 0.0% | 0.0% |
|  | 11A | 2 | 2.7% | 0.2% |
|  | 12F | 1 | 1.4% | 0.1% |
| V116 only  (not PCV15, not PCV20) | 9N | 6 | 8.1% | 0.7% |
|  | 15A | 0 | 0.0% | 0.0% |
|  | 15C | 2 | 2.7% | 0.2% |
|  | 16F | 2 | 2.7% | 0.2% |
|  | 17F | 3 | 4.1% | 0.4% |
|  | 20A | 3 | 4.1% | 0.4% |
|  | 23A | 4 | 5.4% | 0.5% |
|  | 23B | 6 | 8.1% | 0.7% |
|  | 24F | 1 | 1.4% | 0.1% |
|  | 31 | 0 | 0.0% | 0.0% |
|  | 35B | 7 | 9.5% | 0.8% |
| **Total number of serotype detections** | | **74** |  |  |

1. The SSUAD assay for serotype 6A has cross-reactivity with serotype 6C.

## Table S9: Pneumococcal serotype distribution in age group 50-64 years old

Detection of pneumococcal serotypes by SSUAD assays among adults 50-64 years old hospitalized with community-acquired pneumonia. A total of 126 pneumococcal serotypes were detected in 113 unique patients in this age stratum.

| **Age Group: 50 – 64 years old** | | | | |
| --- | --- | --- | --- | --- |
| Conjugate vaccines that include this serotype | Pneumococcal serotype | Patients with serotype detected, n | Percentage of pneumococcal serotype detections (denominator = 126) with this serotype (%) | Percentage of all-cause CAP patients (denominator= 958) with this serotype detected (%) |
| PCV15, PCV20 & V116 | 3 | 21 | 16.7% | 2.2% |
|  | 6A ^a^ | 1 | 0.8% | 0.1% |
|  | 7F | 4 | 3.2% | 0.4% |
|  | 19A | 8 | 6.3% | 0.8% |
|  | 22F | 16 | 12.7% | 1.7% |
|  | 33F | 2 | 1.6% | 0.2% |
| PCV15 & PCV20  (not V116) | 1 | 1 | 0.8% | 0.1% |
|  | 4 | 2 | 1.6% | 0.2% |
|  | 5 | 2 | 1.6% | 0.2% |
|  | 6B | 2 | 1.6% | 0.2% |
|  | 9V | 1 | 0.8% | 0.1% |
|  | 14 | 1 | 0.8% | 0.1% |
|  | 18C | 0 | 0.0% | 0.0% |
|  | 19F | 9 | 7.1% | 0.9% |
|  | 23F | 0 | 0.0% | 0.0% |
| PCV20 & V116  (not PCV15) | 8 | 3 | 2.4% | 0.3% |
|  | 10A | 0 | 0.0% | 0.0% |
|  | 11A | 7 | 5.6% | 0.7% |
|  | 12F | 0 | 0.0% | 0.0% |
| V116 only  (not PCV15, not PCV20) | 9N | 9 | 7.1% | 0.9% |
|  | 15A | 5 | 4.0% | 0.5% |
|  | 15C | 1 | 0.8% | 0.1% |
|  | 16F | 1 | 0.8% | 0.1% |
|  | 17F | 3 | 2.4% | 0.3% |
|  | 20A | 4 | 3.2% | 0.4% |
|  | 23A | 4 | 3.2% | 0.4% |
|  | 23B | 4 | 3.2% | 0.4% |
|  | 24F | 2 | 1.6% | 0.2% |
|  | 31 | 7 | 5.6% | 0.7% |
|  | 35B | 6 | 4.8% | 0.6% |
| **Total number of serotype detections** | | **126** |  |  |

1. The SSUAD assay for serotype 6A has cross-reactivity with serotype 6C.

## Table S10: Pneumococcal serotype distribution in age group ≥65 years old

Detection of pneumococcal serotypes by SSUAD assays among adults ≥65 years old hospitalized with community-acquired pneumonia. A total of 116 pneumococcal serotypes were detected in 101 unique patients in this age stratum.

| **Age Group ≥65 years** | | | | |
| --- | --- | --- | --- | --- |
| Conjugate vaccines that include this serotype | Pneumococcal serotype | Patients with serotype detected, n | Percentage of pneumococcal serotype detections (denominator= 116) with this serotype (%) | Percentage of all-cause CAP patients (denominator= 1107) with this serotype detected (%) |
| PCV15, PCV20 & V116 | 3 | 16 | 13.8% | 1.4% |
|  | 6A ^a^ | 7 | 6.0% | 0.6% |
|  | 7F | 6 | 5.2% | 0.5% |
|  | 19A | 6 | 5.2% | 0.5% |
|  | 22F | 8 | 6.9% | 0.7% |
|  | 33F | 1 | 0.9% | 0.1% |
| PCV15 & PCV20  (not V116) | 1 | 6 | 5.2% | 0.5% |
|  | 4 | 1 | 0.9% | 0.1% |
|  | 5 | 3 | 2.6% | 0.3% |
|  | 6B | 1 | 0.9% | 0.1% |
|  | 9V | 2 | 1.7% | 0.2% |
|  | 14 | 0 | 0.0% | 0.0% |
|  | 18C | 0 | 0.0% | 0.0% |
|  | 19F | 7 | 6.0% | 0.6% |
|  | 23F | 0 | 0.0% | 0.0% |
| PCV20 & V116  (not PCV15) | 8 | 5 | 4.3% | 0.5% |
|  | 10A | 1 | 0.9% | 0.1% |
|  | 11A | 6 | 5.2% | 0.5% |
|  | 12F | 0 | 0.0% | 0.0% |
| V116 only  (not PCV15, not PCV20) | 9N | 3 | 2.6% | 0.3% |
|  | 15A | 0 | 0.0% | 0.0% |
|  | 15C | 3 | 2.6% | 0.3% |
|  | 16F | 6 | 5.2% | 0.5% |
|  | 17F | 5 | 4.3% | 0.5% |
|  | 20A | 2 | 1.7% | 0.2% |
|  | 23A | 8 | 6.9% | 0.7% |
|  | 23B | 3 | 2.6% | 0.3% |
|  | 24F | 0 | 0.0% | 0.0% |
|  | 31 | 4 | 3.4% | 0.4% |
|  | 35B | 6 | 5.2% | 0.5% |
| **Total number of serotype detections** | | **116** |  |  |

- - - - 1. The SSUAD assay for serotype 6A has cross-reactivity with serotype 6C.

## Table S11: Pneumococcal serotype distribution before COVID-19 onset

COVID-19 activity began in the United States in March 2020. This table displays pneumococcal serotype distribution for the study period before onset of COVID-19 activity (September 2018 – February 2020). Pneumococcal serotypes detected by SSUAD assays among adults hospitalized with community-acquired pneumonia are displayed. A total of 209 pneumococcal serotype detections occurred in 189 unique patients.

| **Prior to COVID-19 (September 1, 2018 – February 28, 2020)** | | | | |
| --- | --- | --- | --- | --- |
| Conjugate vaccines that include this serotype | Pneumococcal serotype | Frequency count of serotype detection, n* | Percentage of pneumococcal serotype detections (denominator= 209) due to this serotype (%) | Percentage of all-cause CAP patients (denominator= 1533) with this serotype detected (%) |
| PCV15, PCV20 & V116 | 3 | 32 | 15.3% | 2.1% |
|  | 6A ^a^ | 0 | 0.0% | 0.0% |
|  | 7F | 7 | 3.3% | 0.5% |
|  | 19A | 13 | 6.2% | 0.8% |
|  | 22F | 28 | 13.4% | 1.8% |
|  | 33F | 2 | 1.0% | 0.1% |
| PCV15 & PCV20  (not V116) | 1 | 7 | 3.3% | 0.5% |
|  | 4 | 0 | 0.0% | 0.0% |
|  | 5 | 4 | 1.9% | 0.3% |
|  | 6B | 1 | 0.5% | 0.1% |
|  | 9V | 2 | 1.0% | 0.1% |
|  | 14 | 1 | 0.5% | 0.1% |
|  | 18C | 0 | 0.0% | 0.0% |
|  | 19F | 15 | 7.2% | 1.0% |
|  | 23F | 0 | 0.0% | 0.0% |
| PCV20 & V116  (not PCV15) | 8 | 7 | 3.3% | 0.5% |
|  | 10A | 1 | 0.5% | 0.1% |
|  | 11A | 9 | 4.3% | 0.6% |
|  | 12F | 1 | 0.5% | 0.1% |
| V116 only  (not PCV15, not PCV20) | 9N | 9 | 4.3% | 0.6% |
|  | 15A | 5 | 2.4% | 0.3% |
|  | 15C | 4 | 1.9% | 0.3% |
|  | 16F | 5 | 2.4% | 0.3% |
|  | 17F | 5 | 2.4% | 0.3% |
|  | 20A | 7 | 3.3% | 0.5% |
|  | 23A | 10 | 4.8% | 0.7% |
|  | 23B | 10 | 4.8% | 0.7% |
|  | 24F | 3 | 1.4% | 0.2% |
|  | 31 | 6 | 2.9% | 0.4% |
|  | 35B | 15 | 7.2% | 1.0% |
| **Total number of serotype detections** | | **209** |  |  |

1. The SSUAD assay for serotype 6A has cross-reactivity with serotype 6C.

## Table S12: Pneumococcal serotype distribution during COVID-19 pandemic

COVID-19 activity began in the United States in March 2020. This table displays the pneumococcal serotype distribution for the study period after onset of COVID-19 activity (March 2020 – October 2022, with no enrollment in September – October 2020). Pneumococcal serotypes detected by SSUAD assays among adults hospitalized with community-acquired pneumonia are displayed. A total of 107 pneumococcal serotype detections occurred in 94 unique patients. Pneumococcal serotypes are grouped according to the serotypes contained within three pneumococcal conjugate vaccines (PCV15, PCV20, and V116). Note: Serotype 15B is contained in PCV20 but was not among the 30 serotypes tested in this study and thus not included in this table.

| **During COVID-19 Activity (March 1, 2020 – October 30, 2022)** | | | | |
| --- | --- | --- | --- | --- |
| Conjugate vaccines that include this serotype | Pneumococcal serotype | Frequency count of serotype detection, n | Percentage of pneumococcal serotype detections (denominator= 107) due to this serotype (%) | Percentage of all-cause CAP patients (denominator= 1384) with this serotype detected (%) |
| PCV15, PCV20 & V116 | 3 | 14 | 13.1% | 1.0% |
|  | 6A ^a^ | 10 | 9.3% | 0.7% |
|  | 7F | 4 | 3.7% | 0.3% |
|  | 19A | 9 | 8.4% | 0.7% |
|  | 22F | 3 | 2.8% | 0.2% |
|  | 33F | 2 | 1.9% | 0.1% |
| PCV15 & PCV20  (not V116) | 1 | 0 | 0.0% | 0.0% |
|  | 4 | 3 | 2.8% | 0.2% |
|  | 5 | 3 | 2.8% | 0.2% |
|  | 6B | 2 | 1.9% | 0.1% |
|  | 9V | 4 | 3.7% | 0.3% |
|  | 14 | 0 | 0.0% | 0.0% |
|  | 18C | 0 | 0.0% | 0.0% |
|  | 19F | 2 | 1.9% | 0.1% |
|  | 23F | 0 | 0.0% | 0.0% |
| PCV20 & V116  (not PCV15) | 8 | 4 | 3.7% | 0.3% |
|  | 10A | 0 | 0.0% | 0.0% |
|  | 11A | 6 | 5.6% | 0.4% |
|  | 12F | 0 | 0.0% | 0.0% |
| V116 only  (not PCV15, not PCV20) | 9N | 9 | 8.4% | 0.7% |
|  | 15A | 0 | 0.0% | 0.0% |
|  | 15C | 2 | 1.9% | 0.1% |
|  | 16F | 4 | 3.7% | 0.3% |
|  | 17F | 6 | 5.6% | 0.4% |
|  | 20A | 2 | 1.9% | 0.1% |
|  | 23A | 6 | 5.6% | 0.4% |
|  | 23B | 3 | 2.8% | 0.2% |
|  | 24F | 0 | 0.0% | 0.0% |
|  | 31 | 5 | 4.7% | 0.4% |
|  | 35B | 4 | 3.7% | 0.3% |
| **Total number of serotype detections** | | **107** |  |  |

1. The SSUAD assay for serotype 6A has cross-reactivity with serotype 6C.

## Table S13: Pneumococcal serotype distribution among patients with high-risk conditions

The Centers for Disease Control and Prevention considers people high-risk for pneumococcal infection if they have any of the following conditions: Alcoholism, Cigarette smoking, Cerebrospinal fluid leak, Chronic heart disease (congestive heart failure and cardiomyopathies), Chronic liver disease, Chronic lung disease (COPD and asthma), Cochlear implant, Immunosuppression, Diabetes mellitus. Among 2917 adults hospitalized with CAP in this study, 2275 (78.0%) had at least one of these high-risk conditions. This table shows pneumococcal serotype detections by SSUAD assays among enrolled patients who had at least one high-risk condition. A total of 269 pneumococcal serotype detections occurred in 241 unique patients.

| **Patients with ≥1 high-risk condition for pneumococcal infection** | | | | |
| --- | --- | --- | --- | --- |
| Conjugate vaccines that include this serotype | Pneumococcal serotype | Frequency count of serotype detection, n | Percentage of pneumococcal serotype detections (denominator= 269) due to this serotype (%) | Percentage of all-cause CAP patients (denominator= 2275) with this serotype detected (%) |
| PCV15, PCV20 & V116 | 3 | 37 | 13.8% | 1.6% |
|  | 6A ^a^ | 7 | 2.6% | 0.3% |
|  | 7F | 8 | 3.0% | 0.4% |
|  | 19A | 19 | 7.1% | 0.8% |
|  | 22F | 25 | 9.3% | 1.1% |
|  | 33F | 4 | 1.5% | 0.2% |
| PCV15 & PCV20  (not V116) | 1 | 7 | 2.6% | 0.3% |
|  | 4 | 3 | 1.1% | 0.1% |
|  | 5 | 5 | 1.9% | 0.2% |
|  | 6B | 2 | 0.7% | 0.1% |
|  | 9V | 3 | 1.1% | 0.1% |
|  | 14 | 1 | 0.4% | 0.0% |
|  | 18C | 0 | 0.0% | 0.0% |
|  | 19F | 15 | 5.6% | 0.7% |
|  | 23F | 0 | 0.0% | 0.0% |
| PCV20 & V116  (not PCV15) | 8 | 11 | 4.1% | 0.5% |
|  | 10A | 0 | 0.0% | 0.0% |
|  | 11A | 13 | 4.8% | 0.6% |
|  | 12F | 1 | 0.4% | 0.0% |
| V116 only  (not PCV15, not PCV20) | 9N | 16 | 5.9% | 0.7% |
|  | 15A | 5 | 1.9% | 0.2% |
|  | 15C | 5 | 1.9% | 0.2% |
|  | 16F | 8 | 3.0% | 0.4% |
|  | 17F | 9 | 3.3% | 0.4% |
|  | 20A | 7 | 2.6% | 0.3% |
|  | 23A | 16 | 5.9% | 0.7% |
|  | 23B | 12 | 4.5% | 0.5% |
|  | 24F | 3 | 1.1% | 0.1% |
|  | 31 | 11 | 4.1% | 0.5% |
|  | 35B | 16 | 5.9% | 0.7% |
| **Total number of serotype detections** | | **269** |  |  |

- - - - 1. The SSUAD assay for serotype 6A has cross-reactivity with serotype 6C.

## Table S14: Pneumococcal serotype detections by blood culture and SSUAD

Among 2917 adults hospitalized with CAP in this study, 41 patients had a pneumococcal serotype detected from blood culture. This table shows the serotypes detected by blood culture and by SSUAD for each of these 41 patients. 37 patients had a pneumococcal serotype detected by blood culture that is contained in the 30-serotype SSUAD assay set used in this study. Among these 37 patients, the pneumococcal serotype detected in blood culture was also detected by SSUAD in 31 (83.8%) patients.

| Category | Patient Number | Pneumococcal serotype detected in blood culture | Pneumococcal serotype detected by SSUAD |
| --- | --- | --- | --- |
| SSUAD detected the same serotype that was detected in blood culture, 29/41 (70.7%) | 1 | 3 | 3 |
|  | 2 | 3 | 3 |
|  | 3 | 3 | 3 |
|  | 4 | 3 | 3 |
|  | 5 | 3 | 3 |
|  | 6 | 3 | 3 |
|  | 7 | 6B | 6B |
|  | 8 | 8 | 8 |
|  | 9 | 8 | 8 |
|  | 10 | 8 | 8 |
|  | 11 | 9N | 9N |
|  | 12 | 9N | 9N |
|  | 13 | 9N | 9N |
|  | 14 | 15A | 15A |
|  | 15 | 17F | 17F |
|  | 16 | 19F | 19F |
|  | 17 | 20A | 20A |
|  | 18 | 20A | 20A |
|  | 19 | 20A | 20A |
|  | 20 | 20A | 20A |
|  | 21 | 22F | 22F |
|  | 22 | 22F | 22F |
|  | 23 | 23A | 23A |
|  | 24 | 23A | 23A |
|  | 25 | 31 | 31 |
|  | 26 | 35B | 35B |
|  | 27 | 35B | 35B |
|  | 28 | 35B | 35B |
|  | 29 | 35B | 35B |
| SSUAD detected same serotype detected in blood culture plus one additional serotype, 2/41 (4.9%) | 30 | 3 | 3, 22F |
|  | 31 | 19F | 19F, 33F |
| SSUAD detected a different serotype than blood culture, 1/41 (2.4%) | 32 | 31 | 22F |
| SSUAD negative, blood culture positive for serotype contained in SSUAD set, 5/41 (12.2%) | 33 | 15C | None |
|  | 34 | 22F | None |
|  | 35 | 23A | None |
|  | 36 | 35B | None |
|  | 37 | 35B | None |
| SSUAD negative, blood culture positive for serotype not contained in SSUAD set, 4/41 (9.8%) | 38 | 7C | None |
|  | 39 | 7C | None |
|  | 40 | 35F | None |
|  | 41 | 38 | None |
